# *HER2/neu* Oncogene Silencing in a Breast Cancer Cell Model Using Cationic Lipid-Based Delivery Systems

**DOI:** 10.3390/pharmaceutics15041190

**Published:** 2023-04-08

**Authors:** Adhika Balgobind, Aliscia Daniels, Mario Ariatti, Moganavelli Singh

**Affiliations:** Nano-Gene and Drug Delivery Laboratory, Discipline of Biochemistry, University of KwaZulu-Natal, Private Bag X54001, Durban 4000, South Africa

**Keywords:** cationic liposomes, breast cancer, *HER2/neu*, oncogene, gene silencing, siRNA

## Abstract

The overexpression of the human epidermal growth factor 2 (*HER2/neu*) oncogene is predictive of adverse breast cancer prognosis. Silencing the *HER2/neu* overexpression using siRNA may be an effective treatment strategy. Major requirements for siRNA-based therapy are safe, stable, and efficient delivery systems to channel siRNA into target cells. This study assessed the efficacy of cationic lipid-based systems for the delivery of siRNA. Cationic liposomes were formulated with equimolar ratios of the respective cholesteryl cytofectins, 3β-N-(N′, N′-dimethylaminopropyl)-carbamoyl cholesterol (Chol-T) or N, N-dimethylaminopropylaminylsuccinylcholesterylformylhydrazide (MS09), with the neutral helper lipid, dioleoylphosphatidylethanolamine (DOPE), with and without a polyethylene glycol stabilizer. All cationic liposomes efficiently bound, compacted, and protected the therapeutic siRNA against nuclease degradation. Liposomes and siRNA lipoplexes were spherical, <200 nm in size, with moderate particle size distributions (PDI < 0.4). The siRNA lipoplexes exhibited minimal dose-dependent cytotoxicity and effective *HER2/neu* siRNA transfection in the *HER2/neu* overexpressing SKBR-3 cells. The non-PEGylated Chol-T-siRNA lipoplexes induced the highest *HER2/neu* silencing at the mRNA (10000-fold decrease) and protein levels (>111.6-fold decrease), surpassing that of commercially available Lipofectamine 3000 (4.1-fold reduction in mRNA expression). These cationic liposomes are suitable carriers of *HER2/neu* siRNA for gene silencing in breast cancer.

## 1. Introduction

Millions of people succumb to cancer annually, breast cancer (BC) being the most prevalent and a major cause of cancer death among women. In 2020, 2.3 million women were diagnosed with BC, with 685,000 deaths worldwide [1]. Breast cancer mortality is a greater challenge in developing countries with poor access to medical treatment. Radiation, surgery, and chemotherapy have been the conventional treatment options. Still, despite advances in the formulation of novel chemotherapeutics, their use has been limited due to systemic toxicities, multidrug resistance, and lack of tumor cell specificity [2]. 

Overexpression of the *HER2/neu* oncogene has been found in approximately 30% of all invasive BCs and correlates with more unfettered and destructive tumor growth and greater resistance to cancer chemotherapy [3,4,5]. In *HER2/neu* amplified breast carcinomas, expression levels are significantly higher, varying from 500,000 to more than 2 million receptors per tumor cell, compared to 25,000 to 185,000 receptors per cell in non-amplified tumors [6,7]. The exploitation of the overexpression of the *HER2/neu* oncogene can be a potential therapeutic target for BC. Despite recent advances in treatment strategies, most, if not all, conventional treatments, such as chemotherapy, are limited by a lack of specificity for tumor cells and the cell cycle dependence of many chemotherapeutic agents. This has spurred efforts to develop unique anticancer agents with improved molecular target specificity.

Silencing the *HER/neu* oncogene expression using small interfering RNA (siRNA) may effectively treat patients with *HER2/neu* overexpressing BC. However, the delivery of therapeutic siRNA to intracellular targets for the induction of sequence-specific mRNA degradation creates an encumbrance that limits the success of siRNA therapeutics. These limitations include the polyanionic nature, hydrophilic character, and relatively high molecular weight, which hampers the direct association of the siRNA to the cell membrane, which creates difficulty in the cellular uptake of these molecules by passive diffusion [8,9]. Furthermore, siRNA molecules in the physiological milieu are prone to degradation by serum nucleases, non-targeted biodistribution, and activation of an immune response, limiting its’ systemic applications [5]. Hence, stable, non-toxic nanocarriers are needed to transport therapeutic siRNA efficiently. 

Liposomes have been the most popular of the many lipid-based systems available. Liposomes are vesicles made up of natural membrane constituents and containing an aqueous core. They are biocompatible and biodegradable in vivo, and their by-products are non-toxic [10,11,12], which increased their popularity as efficient siRNA delivery vehicles. Cationic liposomes, due to their positive charge, readily form lipoplexes with the negatively charged siRNA, ensuring favorable intracellular siRNA separation with high transfection and little or no adverse physiological effects [13,14]. The cationic lipids used include a hydrophilic positively charged head group, a lipid hydrophobic tail to anchor the head group to the liposomal membrane bilayer, and a linker to connect the hydrophilic and hydrophobic regions [15,16]. This has led to many novel and interesting cationic lipids being developed over the years [14,17,18,19], including liposomes containing polysaccharides such as chitin and pectin [20]. Of note is the lipid-based siRNA drug partisan (Onpattro), which was approved in 2018 for the treatment of polyneuropathies caused by hereditary transthyretin amyloidosis. This has opened avenues for other siRNA-based nanotherapeutics [21].

When systemically applied, cationic liposomes can activate complement adsorption with plasma proteins [22], resulting in the formation of a protein corona which is recognized and eliminated from circulation by the reticuloendothelial system (RES) or by renal excretion [23]. Hence, liposomes are surface modified by incorporating biocompatible, hydrophilic moieties such as polyethylene glycol (PEG), which provides steric stabilization and ‘masks’ the liposome charge density, preventing opsonization. Hence, the liposome circulation time is increased, and the passive tumor targeting of the liposome is improved [23,24]. However, it was noted that multiple administrations of PEGylated liposomes could lead to increased clearance from the blood, reducing the therapeutic efficiency [25].

This study evaluated the ability of cationic lipid-based carrier systems to deliver target-specific siRNA to breast cancer cells. Cationic cytofectins, 3β-N-(N′, N′-dimethylaminopropyl)-carbamoyl cholesterol (Chol-T) [26] and N, N-dimethylaminopropylaminylsuccinylcholesterylformylhydrazide (MS09) [27] that have been reported previously were used in the liposomal formulation together with DOPE. These liposomes were modified by adding PEG2000 (0–5 mol%) to afford stealth liposomes. The efficacy with which the liposomes-vehiculated siRNA induced silencing of the *HER2/neu* oncogene overexpression in vitro was assessed at both the mRNA and protein levels using qRT-PCR and western blotting, respectively.

## 2. Materials and Methods

### 2.1. Materials

Dioleoylphosphatidylethanolamine (DOPE) and bromophenol blue were obtained from Sigma-Aldrich Chemical Co. (St. Louis, MO, USA). The 1,2-distearoyl-*sn*-glycero-3-phosphoethanolamine-N-[methoxy(polyethylene glycol)-2000] (DSPE-PEG_2000_) was acquired from Avanti Polar Lipids (Alabaster, AL, USA). HyClone^®^ research grade fetal bovine serum (FBS) was purchased from Thermo Scientific (Northumberland, UK). Eagle’s Minimum Essential Medium (EMEM) containing L-glutamine (4.5 g L^−1^), trypsin-EDTA mixture [versene (EDTA) 200 mg L^−1^ and trypsin 170,000 U L^−1^] and antibiotics (100×) containing penicillin G (10,000 U mL^−1^), streptomycin sulfate (10,000 µg mL^−1^) were purchased from Lonza BioWhittaker (Verviers, Liège, Belgium). The human embryonic kidney (HEK293) and breast adenocarcinoma (SKBR-3 and MCF-7) cell lines were sourced directly from the American Type Culture Collection (ATCC) (Manassas, VA, USA). All sterile tissue culture plastic consumables were obtained from Corning Incorporated (Corning, NY, USA). The siGENOME non-targeting siRNA #1 (D-001210-01-20) and ON-TARGETplus SMARTpool, and Human ERBB2 (2064) (L-003126-00-0020) target sequences were obtained from Thermo Scientific (Dharmacon, Lafayette, CO, USA). Agarose, ReadyPrep^TM^ protein extraction kit, 10× Tris/glycine/SDS buffer, 5× transfer buffer, blotting-grade blocker, Tween 20, Trans-Blot^®^ Turbo^TM^ transfer system RTA transfer kit, mini-PROTEAN^®^ TGX^TM^ long shelf life precast gels, 2× Laemmli sample buffer, Precision Plus Protein^TM^ dual extra standards, clarity Western ECL substrate and RT-PCR strip tubes were purchased from Bio-Rad Laboratories (Richmond, CA, USA). TRIzol^®^ Reagent, Lipofectamine^®^ 3000, high capacity cDNA Reverse Transcription Kit with RNase inhibitor, MicroAmp^®^ Fast optical 96-well reaction plates, MicroAmp^®^ optical adhesive films, and DNase/RNase free distilled water were purchased from Life Technologies (Carlsbad, CA, USA). The Neu monoclonal antibody (MW 185 kDa), β-Actin (C4) monoclonal antibody (MW 43 kDa), and goat anti-mouse IgG-HRP secondary antibody were purchased from Santa Cruz Biotechnology, Inc. (Santa Cruz, CA, USA). All other chemicals and general reagents were of analytical grade or higher and purchased from Merck (Darmstadt, Germany) with 18 MΩ water (Milli-Q50) used in preparations.

### 2.2. Liposome Preparation

Cationic liposomes and PEGylated cationic liposomes were prepared using the thin film evaporation method [28]. The two cationic cholesteryl cytofectins (CCC), Chol-T and MS09, were previously synthesized in the laboratory and reported [26,27]. The composition and molar ratio of the liposomal formulations used in the present study are outlined in Table 1. PEGylated liposomes were formulated with 2 or 5 mol% of PEG. The lipid mixture was vortexed and rotor-evaporated (Büchi RE121 Rotavapor, Büchi, Switzerland) to a thin film, which was hydrated overnight at 4 °C in 500 μL sterile HEPES buffered saline (HBS, 20 mM HEPES, 150 mM NaCl; pH 7.5). The sample was then vortexed and sonicated for 5 min at 21 °C and stored at 4 °C.

### 2.3. Liposome: siRNA Complex Preparation

Lipoplexes were freshly prepared by adding the siRNA to varying amounts of the cationic liposome suspensions to obtain different (*w*/*w*) ratios. The reaction mixtures were briefly vortexed and incubated at room temperature for 30 min to allow for lipoplex formation.

### 2.4. Characterization

#### 2.4.1. Transmission Electron Microscopy (TEM)

The morphology of the cationic liposomes and lipoplexes (at optimal binding ratios) was examined by cryo-TEM. Liposomes or lipoplexes (2 µL) were deposited onto a formvar-coated copper grid (Ted Pella Inc., Redding, CA, USA) and contrasted 1:1 (*v*/*v*) with 4% saturated acidic uranyl acetate. The grids were plunged into liquid nitrogen at −180 °C using an injector system (Leica Microsystems EM CPC, Buffalo Grove, IL, USA), and the samples were examined using a JEOL JEM-1010 electron microscope (Jeol, Tokyo, Japan) operating at an accelerating voltage of 100 kV. The images were captured using the associated Soft Imaging System (SIS) MegaView III, bearing a side-mounted 3-megapixel digital camera.

#### 2.4.2. Size, Zeta Potential, and Polydispersity Index

The hydrodynamic size, zeta potential, and polydispersity index (PDI) of the liposomes and lipoplexes were determined by dynamic light scattering (DLS) on a Malvern Nano-ZS ZetaSizer (Malvern Instruments, Worcestershire, UK), equipped with a 5 mW He-Ne laser beam (633 nm, fixed backscattering detection optics positioned at 173°) at 25 °C. All measurements were done in triplicate. All data were analyzed using the ZetaSizer software version 6.30 (Malvern Instruments, Worcestershire, UK).

### 2.5. Band-Shift Assay

This assay was used to determine the optimum binding of the siRNA to the cationic liposomes and prepared as previously described [29]. The siRNA: cationic liposomes (*w/w*) preparations are presented in Table 2, with siRNA kept constant at 0.32 µg. Lipoplexes were loaded into the wells of a 2% agarose gel and electrophoresed at 50 V for 30 min. Gels were visualized, and images were captured on a Vacutec Syngene G: Box BioImaging System (Syngene, Cambridge, UK) using GeneSnap Imaging Software version 7.05 (Syngene, Cambridge, UK). Naked siRNA served as a positive control. 

### 2.6. Serum Nuclease Protection Assay

The siRNA lipoplexes at sub-optimum, optimum, and supra-optimum weight ratios obtained for the band shift assay were used (Table 3). After incubation, the lipoplexes were treated with fetal bovine serum (FBS) to a final concentration of 10% and incubated at 37 °C for 4 h. The reaction was terminated with 10 mM EDTA, and lipoplexes disassociated using 0.5% SDS. After incubation for 20 min at 55 °C, the samples were subjected to agarose gel electrophoresis as in Section 2.5. Control samples included untreated siRNA and FBS-treated siRNA. 

### 2.7. Cell Viability Studies

The cytotoxicity of the lipoplexes was assessed in the HEK293, MCF-7, and SKBR-3 cells using the MTT assay. Cells were seeded at a density of 2.0 × 10^4^ cells per well into 48-well cell plates containing 0.25 mL of complete medium (EMEM supplemented with 10% (*v/v*) FBS and 1% antibiotics) and incubated at 37 °C for 24 h. The siRNA lipoplexes were prepared as in Section 2.3 and Table 3. After incubation, the medium was replaced, and the lipoplexes were added to the cells. The cells were incubated at 37 °C for 48 h, after which the medium was removed and replaced with 25 µL of MTT (5 mg. mL^−1^ in PBS) in 0.25 mL medium. The cells were incubated for an additional 4 h at 37 °C, after which the MTT-containing medium was aspirated, and DMSO (0.2 mL) was added to dissolve the formazan crystals. Untreated cells were used as the positive control (100% cell viability), together with a Lipofectamine^®^ 3000 control at its optimal concentration as per manufacturer’s protocol. All assays were conducted in triplicate. Absorbance was measured at 570 nm using a Vacutec, Mindray MR-96A microplate reader, with DMSO as a blank. The cell viability (%) was then calculated using Equation (1):

% Cell survival (CS) = Average of treated cells/Average of control cells × 100(1)

### 2.8. HER2/neu Silencing at mRNA and Protein Levels

#### 2.8.1. siRNA Transfection

Four different sequences of 19 nucleotides (ON-TARGETplus SMARTpool) were used as potential siRNAs targeting the *HER2/neu* gene. A non-targeting sequence siRNA was used as a non-specific siRNA control. The SKBR-3 cells were seeded into 6-well plates at a density of 1 × 10^5^ cells per well and incubated at 37 °C for 24 h. The medium was then replaced with 1.5 mL fresh complete medium, followed by adding 10 μL of the siRNA lipoplexes (Table 3), with siRNA maintained at 0.64 µg. Controls included untreated cells and Lipofectamine^®^ 3000 (positive transfection control), where lipoplexes were prepared according to the manufacturer’s instructions (5 µL Lipofectamine^®^ 3000 reagent and 2.5 µL siRNA (0.64 µg) in 250 µL EMEM for 5 min at room temperature). All assays were done in triplicate. Post 48 h and 72 h transfection, the cells were harvested for assessing *HER2/neu* gene silencing using qRT-PCR and western blotting, respectively.

#### 2.8.2. RNA Extraction and qRT-PCR

Total cellular RNA from the SKBR-3 cells was extracted using TRIzol^®^ Reagent following the manufacturer’s protocol. The RNA pellet was resuspended in 30 μL RNase-free water. The total RNA was converted into cDNA by reverse transcriptase PCR using the high-capacity cDNA Reverse Transcription (RT) Kit with RNase inhibitor, following the manufacturer’s protocol. Gene expression was quantified by qRT-PCR using the TaqMan^®^ gene expression assays, which are FAM^TM^ dye-labeled and possess a minor-groove binding (MGB) probe. The primers and probe used were the genes of interest *HER2/neu* (Assay ID Hs01001580_m1) and the endogenous control glyceraldehyde 3-phosphate dehydrogenase (*GAPDH*) (Assay ID Hs03929097_g1). Singleplex PCR reactions were conducted as triplicates for all samples. Each reaction mixture (20 μL) contained 10 μL TaqMan^®^ gene expression master mix (AmpliTaq Gold^®^ DNA polymerase, deoxyribonucleotide triphosphates (dNTPs) with deoxyuridine triphosphate (dUTP), UP (Ultra Pure), Uracil-DNA glycosylase (UDG), ROX^TM^ passive reference, as well as buffer components optimized for specificity, sensitivity, and precision), 1 μL 20× TaqMan^®^ gene expression assay mix, and 9 μL sample cDNA. The qRT-PCR was performed under the following conditions: 95 °C for 10 min (hold), followed by 40 cycles of 95 °C for 15 s (denature) and 60 °C for 1 min (anneal/extend) on a BioRad CFX 96^TM^ Real-Time System, C1000 Touch^TM^ Thermal Cycler using CFX Manager Software version 3.0. (Hercules, CA, USA) Relative expression values of *HER2/neu* mRNA normalized to the level of *GAPDH* mRNA were determined using the 2^−∆∆Ct^ method [30].

Fold difference = 2^−∆∆Ct^

∆C_t sample_ − ∆C_t calibrator_ = ∆∆C_t_

C_t GOI_^s^ − C_t norm_ ^s^ = ∆C_t sample_

C_t GOI_^c^ − C_t norm_ ^c^ = ∆C_t calibrator_

where s represents the sample, c the calibrator (untreated cells), GOI the gene of interest *HER2/neu*, and norm the normalizer gene *GAPDH*.

#### 2.8.3. Protein Extraction and Western Blotting

The extraction of total cellular proteins was performed 72 h after transfection, using the ReadyPrepTM Protein Extraction Kit, according to the manufacturer’s specifications (Bio-Rad, Hercules, CA, USA), and quantified using a NanoDrop 2000c spectrophotometer (Thermo Scientific, Wilmington, DE, USA). Approximately 20 μg of protein were treated with an equal volume of 2× Laemmli sample buffer (4% SDS, 10% 2-mercaptoethanol, 20% glycerol, 0.004% bromophenol blue, and 125 mM Tris-HCl, pH 6.8) and heated at 95 °C for 5 min. Before loading, Mini-PROTEAN^®^ TGXTM (10%) long shelf life precast gel cassettes (Bio-Rad Laboratories, Richmond, CA, USA) were placed in a Bio-Rad Mini-PROTEAN^®^ Tetra System, with the upper tank containing chilled 1× Tris/glycine/SDS running buffer (25 mM Tris, 190 mM glycine, and 0.1% (*w*/*v*) SDS, pH 8.3, Bio-Rad Laboratories, Richmond, CA, USA)., and the lower tank filled with chilled 1× Tris/glycine/SDS running buffer. The protein samples were loaded into the wells, and electrophoresis was conducted at room temperature for 30 min at 200 V cm^−1^. A molecular weight marker (3 μL) (Precision Plus ProteinTM dual extra standards) was loaded into the first well. Following electrophoresis, protein transfer (blotting) was conducted using the Bio-Rad Trans-Blot^®^ TurboTM transfer system and RTA transfer kits following the manufacturer’s protocol. The transfer was performed at 2.5 A, 25 V for 10 min to promote high Mw transfer (>150 kDa). The blot and gel were then placed in deionized water. Before antibody incubation, unoccupied sites on the blot were saturated in a solution of 3% blotting-grade blocker (non-fat dry milk) in Tris-buffered saline (20 mM Tris-HCl, pH 7.5, 150 mM NaCl) containing 0.1% Tween 20 (TBST) for 1 h at room temperature. The membranes were incubated overnight at 4 °C in TBST containing either Neu, a mouse monoclonal antibody raised against a synthetic peptide corresponding to amino acids 1242–1255 of human Neu (1:5000) for HER2/neu protein detection, or β-Actin (1:200) used as an internal control for protein loading. The following day, the primary antibody was removed, and the membranes were washed in 20 mL TBST with agitation for 5 min (5×). The membranes were then incubated at room temperature in goat anti-mouse IgG-HRP secondary antibody (1:2000 in TBST). After 1 h, the secondary antibody was decanted, and the membranes were washed in 20 mL TBST with agitation for 5 min (5×). The membranes were developed using a ClarityTM Western ECL substrate kit following the manufacturer’s instructions. The blots were incubated for 5 min at room temperature and then visualized using a Bio-Rad digital imager ChemiDocTM MP system. Band intensity was determined using Image Lab Software version 5.2.2 (BioRad, Hercules, CA, USA).

### 2.9. Statistical Analysis

Data are presented as means ± SD (*n* = 3). Statistical Analysis among mean values was performed using one-way ANOVA followed by the Tukey-Kramer multiple comparisons test between formulations. All statistics were performed using a 95% confidence interval and were considered significant when the *p*-value was less than 0.05 (*p* < 0.05).

## 3. Results

### 3.1. Liposome and Lipoplex Characterization

**Figure 1:**
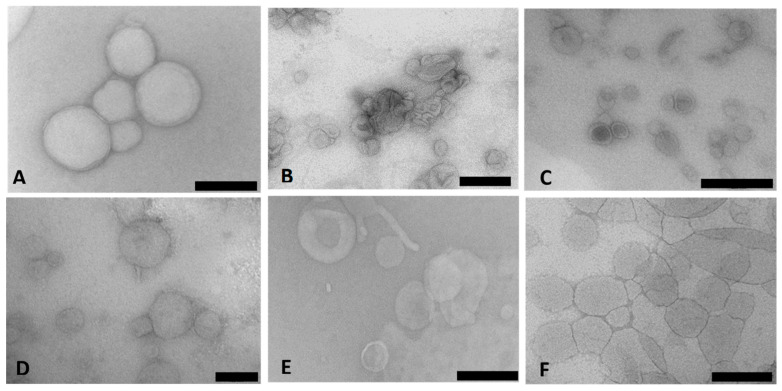
Transmission electron micrographs of cationic liposome: siRNA lipoplexes (N/P (+/−) charge ratios): (**A**) Chol-T; (**B**) Chol-T 2% PEG2000; (**C**) Chol-T 5% PEG2000; (**D**) MS09; (**E**) MS09 2% PEG22000; (**F**) MS09 5% PEG2000. Scale Bar = 100 nm (**D**) or 200 nm (**A**–**C**,**E**,**F**).

Cryo-TEM enabled the direct examination of the ultrastructure of the colloidal carriers in their frozen-hydrated state. Figure 1A–F presents the TEM micrographs of the Chol-T and MS09 lipoplexes with siRNA. The images revealed a heterogeneous population of typically spherical or ellipsoidal structures with a bilayered membrane surrounding the internal aqueous core. The lipoplexes appeared well dispersed with little aggregation. 

DLS measurements revealed nanometer (nm) sized particles which were accompanied by moderate to narrow polydispersity indices (PDI) and good zeta (ζ) potentials, as summarized in Table 4. For Chol-T, sizes ranged from 71.64 nm to 121.07 nm, while the lipoplexes ranged from 149.61 nm to 187.97 nm. The MS09 liposomes and lipoplexes were slightly smaller (65.47 nm to 113.02 nm and 103.24 nm to 169.13 nm, respectively). The MS09-based lipoplexes also exhibited better zeta potentials (16.08 mV to 53.21 mV) than the Chol-T-based lipoplexes. For both liposomes, a size reduction upon functionalization was noted. Furthermore, functionalization with PEG reduced the zeta potential, especially in the Chol-T-based liposomes and lipoplexes.

### 3.2. Liposome Binding and Protection Efficiencies

**Figure 2:**
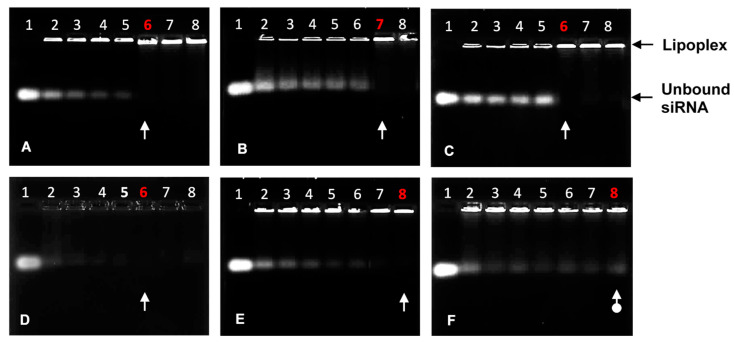
Band-shift assay showing the binding interaction between varying amounts of PEGylated and non-PEGylated cationic liposomes with siRNA (0.32 μg). (**A**) Chol-T: Lanes 1–8 (0, 3.20, 3.52, 3.84, 4.16, 4.48, 4.80, and 5.12 μg); (**B**) Chol-T 2% PEG2000: Lanes 1–8 (0, 6.08, 6.40, 6.72, 7.04, 7.36, 7.68, and 8.00 μg); (**C**) Chol-T 5% PEG2000: Lanes 1–8 (0, 10.56, 10.88, 11.20, 11.52, 11.84, 12.16, and 12.48 μg); (**D**) MS09: Lanes 1–8 (0, 5.44, 5.76, 6.08, 6.40, 6.72, 7.04, and 7.36 μg); (**E**) MS09 2% PEG2000: Lanes 1–8 (0, 6.08, 6.40, 6.72, 7.04, 7.36, 7.68, and 8.00 μg); (**F**) MS09 5% PEG2000: Lanes 1–8 (0, 9.60, 9.92, 10.24, 10.56, 10.88, 11.20, and 11.52 μg). White arrows indicate endpoint ratios, except in 2F, where it indicates no clear endpoint.

The agarose gel migration patterns illustrated that all liposomal formulations effectively bound the siRNA, as seen in Figure 2. The complete or endpoint binding ratios of the siRNA by the cationic liposomes are represented in Table 5. Ratios below the optimum exhibited bands that fluoresced brightly and then gradually lightened until the band completely disappeared, signifying that the negatively charged siRNA was neutralized entirely at those respective N/P charge ratios.

**Figure 3:**
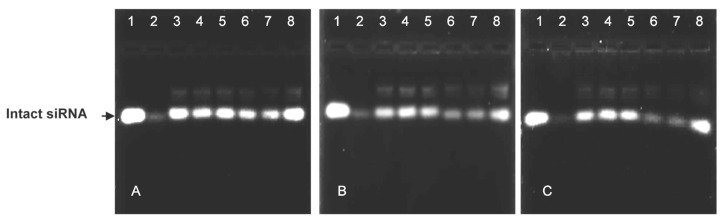
Nuclease protection assay of cationic and PEGylated cationic liposome-siRNA complexes in the presence of 10% FBS. Reaction mixtures (10 μL) contained siRNA (0.2 μg) and varying amounts of liposome suspension. (**A**) Lanes 3–5: Chol-T (2.4, 2.8, 3.2 μg) Lanes 6–8: MS09 (3.8, 4.2, 4.6 μg); (**B**) Lanes 3–5: Chol-T 2% PEG_2000_ (4.4, 4.8, 5.2 μg), Lanes 6–8: Chol-T 5% PEG_2000_ (7.0, 7.4, 7.8 μg); (**C**) Lanes 3–5: MS09 2% PEG_2000_ (4.6, 5.0, 5.4 μg), Lanes 6–8: MS09 5% PEG_2000_ (6.8, 7.2, 7.6 μg). Lane 1: FBS-untreated naked siRNA (0.2 μg) and lane 2: FBS-treated siRNA (0.2 μg).

The nuclease protection assay showed the ability of the cationic liposomes to protect their siRNA cargo from degradation (Figure 3). The positive controls (lanes 1) and negative controls (lanes 2) assessed the degree of serum nuclease digestion of the lipoplexes. Both non-PEGylated lipoplexes offered better protection to their siRNA cargo than their PEGylated counterparts (Figure 3A). 

### 3.3. Cell Viability Assay

**Figure 4:**
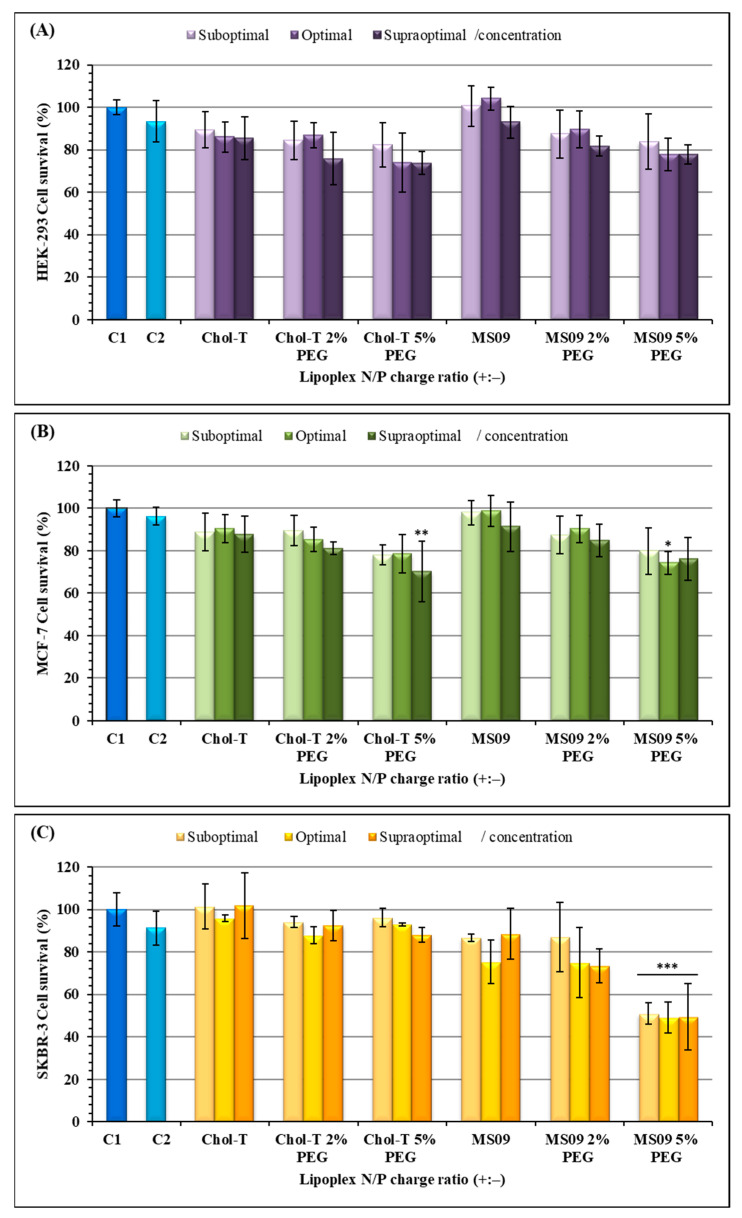
Cell viability studies of siRNA lipoplexes in (**A**) HEK293, (**B**) MCF-7, and (**C**) SKBR-3 cells in vitro. Incubation mixtures contained 0.32 μg of siRNA with varying amounts of liposome from suboptimal to supraoptimal N/P (+:−) charge ratios: Chol-T (3.4, 3.9, 4.4); Chol-T 2% PEG (5.8, 6.3, 6.8); Chol-T 5% PEG (8.3, 8.8, 9.3); MS09 (4.9, 5.4, 5.9); MS09 2% PEG (5.5, 6.0, 6.5); MS09 5% PEG (7.4, 7.9, 8.4). C1 = cells only- untreated), and C2 = Lipofectamine3000 treated cells. Data are presented as means ± SD (n = 3). * *p* < 0.05, ** *p* < 0.01, and *** *p* < 0.001 were considered statistically significant vs. the cell control.

The 3-(4, 5-dimethyl-2-thiazolyl)-2, 5-diphenyl-2H-tetrazolium bromide (MTT) assay was used to assess the viability of cells exposed to the transfection complexes (Figure 4). All cells maintained a viability of over 70%, except for the SKBR-3 cells exposed to MS09 5% PEG2000 lipoplexes which showed a 50% decrease in cell viability (*p* < 0.001). Figure 4A,B shows that for the HEK293 and MCF-7 cells, a dose-dependent cytotoxic effect with an increase in charge ratios was observed. The MS09-based lipoplexes were slightly less toxic than the Chol-T -based lipoplexes in the HEK293 cells (Figure 4A). The MS09: siRNA lipoplexes at N/P ratios of 4.9 and 5.4 were non-toxic to the HEK293 cells and produced improved cell viability (>100%). In the SKBR-3 cells, a dose-independent decrease in cell viability was observed for the MS09-based lipoplexes, and in some instances, MS09 was slightly more toxic than Chol-T. This was seen for the MS09 lipoplex containing 5% PEG, where cell viability was slightly above 50%. Nevertheless, all other lipoplexes produced cell viability above 70%.

### 3.4. HER2/neu Gene Silencing in SKBR-3 (Breast Cancer) Cells

#### 3.4.1. Real-Time Quantitative PCR (qRT-PCR)

**Figure 5:**
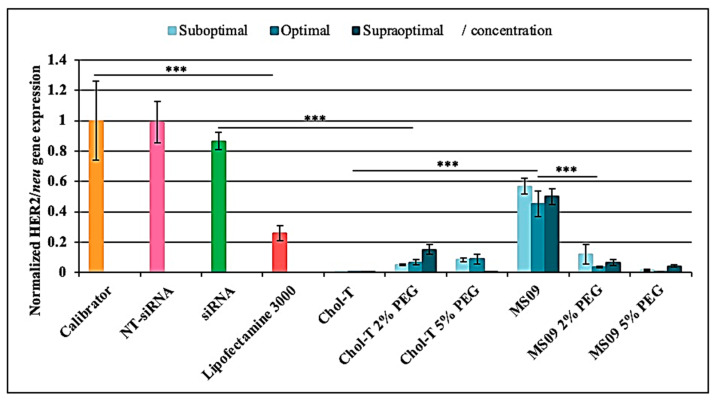
Analysis of *HER2/neu* gene expression in SKBR-3 cells by qRT-PCR. The vertical axis represents the relative quantification of *HER2/neu* normalized against *GAPDH* mRNA level using the comparative quantification algorithm 2-∆∆Ct [30]. The mean ± SD of independent experiments (n = 3). *** *p* < 0.001 was considered statistically significant.

According to the qRT-PCR results depicted in Figure 5, the calibrator revealed high *HER2/neu* gene levels. The NT-siRNA showed no knockdown, whereas the uncomplexed *HER2/neu* target siRNA resulted in a slight decrease of the *HER2/neu* gene at the mRNA level. The gene expression levels of the housekeeping normalizer gene *GAPDH* did not differ significantly among the control and test samples. Each liposomal formulation could efficiently deliver the siRNA against the *HER2/neu* oncogene in the SKBR-3 BC cells in the presence of serum, as indicated by the downregulation of *HER2/neu* at the mRNA level (*p* < 0.001). A comparison of the non-PEGylated Chol-T and MS09 lipoplexes at the different charge ratios revealed that Chol-T:siRNA lipoplexes induced the highest *HER2/neu* silencing effect at all concentrations (*p* < 0.001), exceeding that of Lipofectamine^®^-3000 (4.1- fold decrease).

#### 3.4.2. HER2/neu Protein Expression 

**Figure 6:**
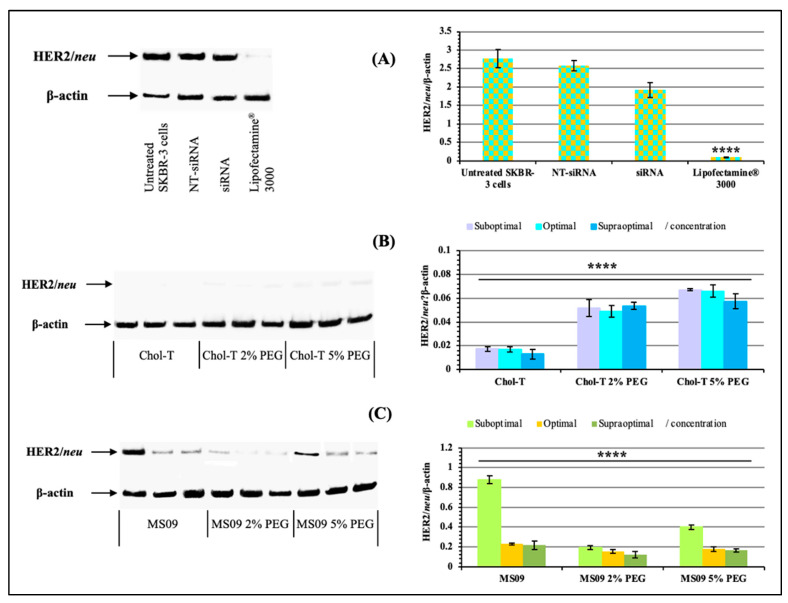
Analysis of HER2/*neu* oncoprotein expression by western blotting. (**A**) Non-treated SKBR-3 cells, NT-siRNA (non-targeting siRNA), and siRNA (*HER2/neu* targeting siRNA alone) served as negative controls. Lipofectamine^®^ 3000-siRNA was included as a positive control, (**B**) SKBR-3 cells were treated with *HER2/neu* target siRNA: PEGylated and non-PEGylated Chol-T lipoplexes and (**C**) SKBR-3 cells were treated with HER2/*neu* target siRNA: PEGylated and non-PEGylated MS09 lipoplexes. HER2/*neu* receptor expression was determined in cellular lysates by Western blotting analysis using the HER2/*neu* and β-actin antibodies. Graphs represent the HER2/*neu*/β-actin normalization ratios. Data are presented as means ± SD (n = 3). **** *p* < 0.0001 is considered statistically significant vs. the untreated SKBR-3 cell control.

The HER2/neu protein expression levels were analyzed using western blotting. Results were analyzed based on densitometric values, and β-actin control was used to determine the normalization or equivalency of lane loading. High levels of HER2/neu protein expression (ratios) in negative control treatments relative to β-actin (Figure 6A) were observed. In contrast, dramatic decreases in HER2/neu protein expression levels were observed for all the siRNA delivery systems employed in this study.

**Figure 7:**
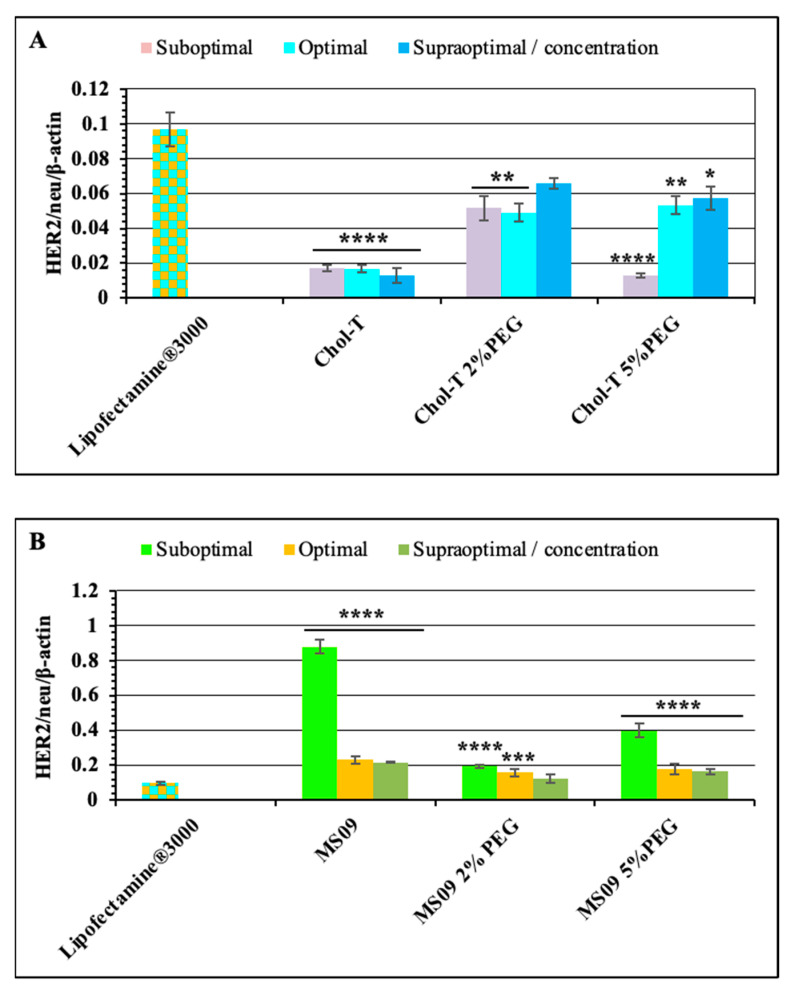
Comparison of HER2/neu oncogene knockdown of PEGylated and non-PEGylated (**A**) Chol-T and (**B**) MS09 lipoplexes vs. Lipofectamine^®^ 3000 following Western Blotting Analysis. Graphs represent the HER2/neu/β-actin normalization ratios. Data are presented as means ± SD (n = 3). * *p* < 0.1, ** *p* < 0.01, *** *p* < 0.001 and **** *p* < 0.0001 are considered statistically significant vs. Lipofectamine^®^ 3000.

The non-PEGylated Chol-T and MS09 liposomes resulted in a dose-dependent decrease in HER2/neu protein expression, with an increase in the N/P charge ratio causing a reduction in protein expression. Chol-T lipoplexes exhibited a higher decrease in protein expression compared to the MS09 lipoplexes. Normalized HER2/neu protein expression levels decreased by: 53.50, 56.48, and 51.89-fold (Chol-T 2% PEG2000); 41.29, 41.96, and 48.28 (Chol-T 5% PEG2000); 14.18, 17.60 and 22.61 (MS09 2% PEG2000); 6.94, 15.55 and 16.68 (MS09 5% PEG2000) compared to the untreated SKBR-3 cells. Figure 7A further highlights the significant silencing of the *HER2/neu* gene in cells treated with Chol-T nanocomplexes against the Lipofectamine^®^ control. This knockdown is also much greater than that for the MS09 lipoplexes (Figure 7B).

## 4. Discussion

Both cationic liposomes were previously synthesized and characterized, and their complexes with DNA were described as clusters of spherical liposomes [26,27], as observed in the current study. However, some variations were noted, which could be due to the amorphous nature or flexibility of the liposomal membranes. The surface modifications of the liposomal vesicles with PEG chains are not clear under TEM due to the low contrast of PEG [31].

The size of the liposome influences its cellular interaction and uptake, with sizes of 50–250 nm proposed to bypass elimination and clearance by the RES [32,33]. Our results indicate that the liposomes were relatively stable and homogeneous and are within the size range generally considered ideal for both cellular uptake and systematic circulation [34,35]. Lipoplex formation resulted in a size increase (up to 187.97 nm), possibly due to the binding of the siRNA onto the liposome surface compared to its’ entrapment within the liposomes. The decrease in size and PDI of the Chol-T and MS09 liposomes upon PEGylation may be attributed to the strong inter-bilayer repulsion that can overcome the attractive van der Waals forces, thus providing a steric barrier at the surface of the liposomes. PEG chains are reported to provide steric stabilization, prevent vesicle aggregation and encourage the formation of homogeneous, smaller liposomes with good colloidal stability and biocompatibility [36,37]. The zeta potential indicates the colloidal stability with values < −25 mV or > +25 mV considered stable. The zeta potentials of the liposomes and lipoplexes decreased upon the addition of PEG due to the masking of the positive charges. However, liposomes containing 2% PEG remained highly positive (>20 mV), indicating that the amount, length, and density of the PEG chains can affect the degree of stability offered [38]. It is important that the amount of positive charge present be sufficient to allow for siRNA binding and endosomal escape without affecting its disassociation from the lipoplex [39]. The successful transfection noted confirms the favorable properties of these liposomes. The MS09 5% PEG had higher PDI values, suggesting a slightly polydisperse liposomal population. It was proposed that for liposomes, a PDI of 0.3 is acceptable and indicates a homogenous population of vesicles [40].

More of the PEGylated cationic liposomes were required to completely bind the siRNA, possibly due to the partial masking of the positive charges by PEG. Although the 5% MS09 liposomes could retard the siRNA, no clear endpoint was established. Overall, the liposomes showed good protection of the siRNA in the presence of FBS, with the 5% PEG liposomes offering the least protection. Turetskiy and Coworkers (2017) observed sandwich-like structures for the siRNA lipoplexes and proposed that this improved their ability to protect the nucleic acid from degradation [41]. PEGylated liposomes were proposed to form looser complexes with siRNA, with PEG chains preventing the electrostatic interaction between the siRNA and the cationic lipid [42]. This could lead to the siRNA being exposed on the liposome’s surface and easily attacked by nucleases present in serum. There was also some correlation with the zeta potentials obtained, indicating that liposomal instability can affect the ability of the carrier to protect its cargo from nucleases. A similar serum nuclease digestion profile was reported for DOTAP-based cationic liposomes complexed with a TNF-α-siRNA [43]. 

The low cytotoxicity observed for these lipoplexes could be attributed to monovalent cationic lipids, which are less toxic than multivalent cationic lipids [44]. Furthermore, it was suggested that monovalent cationic liposomes possess good gene knockout due to their favorable cellular uptake and endosomal escape due to the ion-pair mechanism [25]. The DOPE and cholesterol in the liposomal formulations contributed to the lipoplexes biocompatibility and stability, with cholesterol, a natural biomembrane component known to regulate the fluidity of the lipid bilayer [15,45]. These results are similar to previous reports where an increase in the length of the spacer segment resulted in reduced cytotoxicity in vitro [46]. Moreover, lipid-containing carbamate bonds have lower cytotoxicity, are stable under neutral conditions, and are prone to hydrolysis at low pH (in the endosome) [47,48,49]. The increased cytotoxicity exhibited by the PEGylated lipoplexes was proposed to be due to a higher cationic lipid concentration [50] and the possibility that these lipids generate reactive oxygen species (ROS). Since cancer cells exhibit greater ROS stress than normal cells [51,52], the SKBR-3 cell viability may have affected ROS production. 

PEG-based Chol-T lipoplexes produced a reduced HER2/neu mRNA expression, which is in accordance with a previous report where PEGylation of DC-Chol/DOPE siRNA lipoplexes caused a decrease in size and zeta potential, and no HER2 silencing in SKBR-3 cells [50]. However, PEG-based MS09 lipoplexes significantly increased the *HER2/neu* gene silencing (*p* < 0.001) despite a size and zeta potential reduction. This may be related to their slightly lower cell viability. The HER2/*neu* protein expression results corroborated the qRT-PCR findings, suggesting that cationic liposomes bearing shorter aliphatic chains offer better transfection efficiency. It was proposed that increased transfection efficiency with shorter hydrocarbon chains is due to increased bilayer fluidity, leading to enhanced inter-membrane transfer and mixing of the lipid membrane [53]. Although the MS09 lipoplexes did not elicit *HER2/neu* gene silencing at the same level as the Chol-T lipoplexes, they were still effective. These MS09 liposomes have previously shown significant *c-MYC* silencing in MCF-7 and HT-29 cells, surpassing that of Lipofectamine^®^-3000 [54]. Cationic liposomes have also been successfully used to deliver siRNA (siPlk1) to breast cancer cells in vitro [55].

Despite advances in HER2-targeted monoclonal antibody therapy [56], including the use of Trastuzumab or Herceptin [57], the need for chemotherapy is still crucial for breast cancer patients. In addition, the approval of the siRNA drug, Onpattro and its effectiveness in improving nerve function has highlighted the immense potential of RNA interference for the treatment of other disorders. Hence, nanoparticle-mediated gene silencing may be a viable option. Overall, findings from this study encourage future studies and optimizations of these cationic liposomes for their use in gene silencing in cancer. 

## 5. Conclusions

This study highlights the immense potential of both PEGylated and non-PEGylated Chol-T and MS09 cationic liposomes in *HER2/neu* siRNA oncogene silencing in SKBR-3 cells. The findings support the hypothesis that these non-viral cationic liposome systems can facilitate future-therapeutic siRNA gene delivery. However, with the caveat that PEGylated cationic liposomes be used with caution since their effects varied with each of the cytofectins used in this study. Post-inserting PEG on preformed siRNA lipoplexes is conceivably a viable option to alleviate some of the adverse effects of using PEG. This strategy demonstrates better siRNA binding and the potential for masking siRNAs and protecting them from nuclease digestion. Among the cationic liposomes utilized in this study, non-PEGylated Chol-T lipoplexes induced the highest gene silencing at all tested N/P ratios, as indicated by the significant decrease in gene expression (10,000-fold decrease, *p* < 0.001). They exceeded the silencing effect obtained by Lipofectamine^®^-3000 (4.1-fold reduction). Chol-T lipoplexes were well dispersed, colloidally stable, possessed the lowest N/P charge ratio, and exhibited minimal cytotoxicity in the SKBR-3 cells, which may have been attributed to their superior transfection. Given their biocompatibility and excellent transfection efficiency compared to the commercial transfection agent, these cationic liposomes offer a promising and attractive alternative for siRNA gene therapy in vivo. In addition, a combination therapy using siRNA and an anticancer drug can be employed as a further study.

## Figures and Tables

**Table 1:** Composition and molar ratios of the different cationic liposomal formulations.

Liposomal Formulation	Molar Ratios of the Respective Cationic Liposome Components (μmol/500 μL)			Total Lipid Content (µg/µL)
	Cytofectin	DOPE	PEG	
Chol-T: DOPE	1.00	1.00	-	2.517
Chol-T:DOPE:2% PEG	1.00	0.96	0.04	2.648
Chol-T:DOPE:5% PEG	1.00	0.90	0.10	2.938
MS09:DOPE	1.00	1.00	-	2.746
MS09:DOPE:2% PEG	1.00	0.96	0.04	2.914
MS09:DOPE:5% PEG	1.00	0.90	0.10	3.168

**Table 2:** Varying amounts (µg) of PEGylated and non-PEGylated cationic liposomes were added to siRNA for the band shift assay. Columns 1–8 correspond to the lanes of the agarose gel.

Components	Mass (µg)							
	1	2	3	4	5	6	7	8
Chol-T:DOPE	0	3.20	3.52	3.84	4.16	4.48 *	4.80	5.12
Chol-T:DOPE:2% PEG	0	6.08	6.40	6.72	7.04	7.36	7.68 *	8.00
Chol-T:DOPE:5% PEG	0	10.56	10.88	11.20	11.52	11.84 *	12.16	12.48
MS09:DOPE	0	5.44	5.76	6.08	6.40	6.72 *	7.04	7.36
MS09:DOPE:2% PEG	0	6.08	6.40	6.72	7.04	7.36	7.68	8.00 *
MS09:DOPE:5% PEG	0	9.60	9.92	10.24	10.56	10.88	11.20	11.52 *

Endpoints are indicated by an *. Column 1 (lane 1) = siRNA in the absence of cationic liposomes.

**Table 3:** Lipoplexes at the sub-optimum, optimum and supra-optimum ratios (*w/w*).

Liposomal Formulation	Sub-Optimum Ratio (*w/w*)	Optimum Ratio (*w/w*)	Supra-Optimum Ratio (*w/w*)
Chol-T	1:12	1:14	1:16
Chol-T 2% PEG_2000_	1:22	1:24	1:26
Chol-T 5% PEG_2000_	1:35	1:37	1:39
MS09	1:19	1:21	1:23
MS09 2% PEG_2000_	1:23	1:25	1:27
MS09 5% PEG_2000_	1:34	1:36	1:38

Note: siRNA was kept constant at 0.2 µg.

**Table 4:** Size, zeta (**ζ**) potential, and polydispersity indices (PDI) of the cationic liposomes and their complexes with siRNA at their optimum binding ratios (*w/w*).

Cationic Liposome	Liposome			siRNA Lipoplex		
	Size (nm)	ζ Potential (mV) ± SD	PDI	Size (nm)	ζ Potential (mV) ± SD	PDI
Chol-T	121.07 ± 11.8	44.09 ± 10.56	0.321	187.97 ± 12.9	47.26 ± 5.39	0.127
Chol-T 2% PEG	71.64 ± 2.6	32.51 ± 11.02	0.304	149.61 ± 10.5	39.05 ± 8.16	0.227
Chol-T 5% PEG	74.18 ± 1.9	–1.12 ± 4.818	0.136	153.24 ± 18.9	9.78 ± 1.13	0.109
MS09	113.02 ± 13.5	53.21 ± 4.329	0.348	169.13 ± 19.2	44.61 ± 7.56	0.326
MS09 2% PEG	66.68 ± 1.7	39.43 ± 1.185	0.136	103.24 ± 9.1	20.88 ± 3.052	0.218
MS09 5% PEG	65.47 ±1.3	16.08 ± 3.799	0.269	138.64 ± 10.2	41.26 ± 9.79	0.337

**Table 5:** Band-shift assay endpoints and charge ratios of the various cationic/PEGylated cationic liposomes.

Cationic Liposome	siRNA: Cationic Liposome Endpoint (*w/w*)	NP Charge Ratio (+/−)
Chol-T	1:14	1:3.9
Chol-T 2% PEG_2000_	1:24	1:6.3
Chol-T 5% PEG_2000_	1:37	1:8.8
MS09	1:21	1:5.4
MS09 2% PEG_2000_	1:25	1:6.0
MS09 5% PEG_2000_	1:36	1:7.9

## Data Availability

The data and contributions presented in the study are included in the article. Further inquiries can be directed to the corresponding author.

## References

[1] World Health Organization Breast Cancer. https://www.who.int/news-room/fact-sheets/detail/breast-cancer..

[2] Rizvi S.A., Saleh A.M. (2018). Applications of nanoparticle systems in drug delivery technology. Saudi Pharm. J..

[3] Menard S., Tagliabue E., Campiglio M., Pupa S.M. (2000). Role of HER2 gene overexpression in breast carcinoma. J. Cell. Physiol..

[4] Núñez C., Capelo J.L., Igrejas G., Alfonso A., Botana L.M., Lodeiro C. (2016). An overview of the effective combination therapies for the treatment of breast cancer. Biomaterials.

[5] Padayachee J., Daniels A., Balgobind A., Ariatti M., Singh M. (2020). HER2/neu and MYC Gene Silencing in breast cancer: Therapeutic potential and advancement in non-viral nanocarrier systems. Nanomedicine.

[6] Tsé C., Gauchez A.S., Jacot W., Lamy P.J. (2012). HER2 shedding and serum HER2 extracellular domain: Biology and clinical utility in breast cancer. Cancer Treat. Rev..

[7] Yarden Y. (2001). Biology of HER2 and its importance in breast cancer. Oncology.

[8] Whitehead K.A., Langer R., Anderson D.G. (2009). Knocking down barriers: Advances in siRNA delivery. Nat. Rev. Drug Discov..

[9] Daniels A., Singh M. (2019). Sterically stabilized siRNA: Gold nanocomplexes enhance c-MYC in a breast cancer cell model. Nanomedicine.

[10] Kesharwani P., Gajbhiye V., Jain N.K. (2012). A review of nanocarriers for the delivery of small interfering RNA. Biomaterials.

[11] Oh Y.K., Park T.G. (2009). siRNA delivery systems for cancer treatment. Adv. Drug Deliv. Rev..

[12] Shim G., Kim M.G., Park J.Y., Oh Y.K. (2013). Application of cationic liposomes for delivery of nucleic acids. Asian, J. Pharm. Sci..

[13] Li M., Li S., Li Y., Yang G., Li M., Xie Y., Su W., Wu J., Ma W., Li H. (2022). Cationic liposomes co-deliver chemotherapeutics and siRNA for the treatment of breast cancer. Eur. J. Med. Chem..

[14] Vaidya S., Jeengar M.K., Wadaan M.A., Mahboob S., Kumar P., Reece L.M., Bathula S.R., Dutta M. (2022). Design and In Vitro Evaluation of Novel Cationic Lipids for siRNA Delivery in Breast Cancer Cell Lines. Evid.-Based Complement. Altern. Med..

[15] Tenchov R., Bird R., Curtze A.E., Zhou Q. (2021). Lipid Nanoparticles-From Liposomes to mRNA Vaccine Delivery, a Landscape of Research Diversity and Advancement. ACS Nano..

[16] Gao H., Hui K.M. (2001). Synthesis of a novel series of cationic lipids that can act as efficient gene delivery vehicles through systematic heterocyclic substitution of cholesterol derivatives. Gene Ther..

[17] Elsana H., Olusanya T.O.B., Carr-wilkinson J., Darby S., Faheem A., Elkordy A.A. (2019). Evaluation of novel cationic gene based liposomes with cyclodextrin prepared by thin film hydration and microfluidic systems. Sci. Rep..

[18] Hattori Y., Tang M., Torii S., Tomita K., Sagawa A., Inoue N., Yamagishi R., Ozaki K.-I. (2022). Optimal combination of cationic lipid and phospholipid in cationic liposomes for gene knockdown in breast cancer cells and mouse lung using siRNA lipoplexes. Mol. Med. Rep..

[19] Domínguez-Arca V., Sabín J., García-Río L., Bastos M., Taboada P., Barbosa S., Prieto G. (2022). On the structure and stability of novel cationic DPPC liposomes doped with gemini surfactants. J. Mol. Liq..

[20] Corrie L., Gulati M., Awasthi A., Vishwas S., Kaur J., Khursheed R., Porwal O., Alam A., Parveen S.R., Singh H. (2022). Harnessing the dual role of polysaccharides in treating gastrointestinal diseases: As therapeutics and polymers for drug delivery. Chem.-Bio.Interact..

[21] Akinc A., Maier M.A., Manoharan M., Fitzgerald K., Jayaraman M., Barros S., Ansell S., Du X., Hope M.J., Madden T.D. (2019). The Onpattro story and the clinical translation of nanomedicines containing nucleic acid-based drugs. Nat. Nanotechnol..

[22] Caracciolo G. (2015). Liposome-protein corona in a physiological environment: Challenges and opportunities for targeted delivery of nanomedicines. Nanomed. Nanotechnol. Biol. Med..

[23] Sercombe L., Veerati T., Moheimani F., Wu S.Y., Sood A.K., Hua S. (2015). Advances and Challenges of Liposome Assisted Drug Delivery. Front. Pharmacol..

[24] Gaumet M., Vargas A., Gurny R., Delie F. (2008). Nanoparticles for drug delivery: The need for precision in reporting particle size parameters. Eur. J. Pharm. Biopharm..

[25] Zhen S., Li X. (2020). Liposomal delivery of CRISPR/Cas9. Cancer Gene Ther..

[26] Singh M., Ariatti M. (2003). Targeted gene delivery into HepG2 cells using complexes containing DNA, cationized asialoorosomucoid and activated cationic liposomes. J. Control. Release.

[27] Singh M., Ariatti M. (2006). A cationic cytofectin with long spacer mediates favourable transfection in transformed human epithelial cells. Int. J. Pharm..

[28] Gao X., Huang L. (1991). A novel cationic liposome reagent for efficient transfection of mammalian cells. Biochem. Biophys. Res. Commun..

[29] Singh M., Narayanan K. (2021). Assessing nucleic acid: Cationic nanoparticle interaction for gene delivery. Bio-Carrier Vectors.

[30] Livak K.J., Schmittgen T.D. (2001). Analysis of relative gene expression data using real-time quantitative PCR and the 2^-∆∆CT^ method. Methods.

[31] Kuntsche J., Horst J.C., Bunjes H. (2011). Cryogenic transmission electron microscopy (cryo-TEM) for studying the morphology of colloidal drug delivery systems. Int. J. Pharm..

[32] Lorenzer C., Dirin M., Winkler A.M., Baumann V., Winkler J. (2015). Going beyond the liver: Progress and challenges of targeted delivery of siRNA therapeutics. J. Control. Release.

[33] Resnier P., Montier T., Mathieu V., Benoit J.P., Passirani. C. (2013). A review of the current status of siRNA nanomedicines in the treatment of cancer. Biomaterials.

[34] Kapoor M., Burgess D.J., Patil S.D. (2012). Physicochemical characterization techniques for lipid-based delivery systems for siRNA. Int. J. Pharm..

[35] Mével M., Kamaly N., Carmona S., Oliver M.H., Jorgensen M.R., Crowther C., Salazar F.H., Marion P.L., Fujino M., Natori Y. (2010). DODAG; a versatile new cationic lipid that mediates efficient delivery of pDNA and siRNA. J. Control. Release.

[36] Kenworthy A.K., Hristova K., Needham D., McIntosh T.J. (1995). Range and magnitude of the steric pressure between bilayers containing phospholipids with covalently attached poly(ethylene glycol). Biophys. J..

[37] Needham D., McIntosh T.J., Lasic D.D. (1992). Repulsive interactions and mechanical stability of polymer-grafted lipid membranes. Biochim. Biophys. Acta..

[38] Hassan S., Prakash G., Ozturk A.B., Saghazadeh S., Sohail M.F., Seo J., Dokmeci M.R., Zhang Y.S., Khademhosseini A. (2017). Evolution and Clinical Translation of Drug Delivery Nanomaterials. Nano Today.

[39] Liu C., Zhang L., Zhu W., Guo R., Sun H., Chen X., Deng N. (2020). Barriers and Strategies of Cationic Liposomes for Cancer Gene Therapy. Mol. Ther.-Methods Clin. Dev..

[40] Danaei M., Dehghankhold M., Ataei S., Davarani F.H., Javanmard R., Dokhani A., Khorasani S., Mozafari M.R. (2018). Impact of Particle Size and Polydispersity Index on the Clinical Applications of Lipidic Nanocarrier Systems. Pharmaceutics.

[41] Turetskiy E.A., Koloskova O.O., Nosova A.S., Shilovskiy I.P., Sebyakin Y.L., Khaitov M.R. (2017). Physicochemical properties of lipopeptide-based liposomes and theircomplexes with siRNA. Biomed. Khim..

[42] Belletti D., Tosi G., Forni F., Lagreca I., Barozzi P., Pederzoli F., Vandelli M.A., Riva G., Luppi M., Ruozi B. (2016). PEGylated siRNA lipoplexes for silencing of BLIMP-1 in Primary Effusion Lymphoma: In vitro evidences of antitumoral activity. Eur. J. Pharm. Biopharm..

[43] Pandi P., Jain A., Kommineni N., Ionov M., Bryszewska M., Khan W. (2018). Dendrimer as a new potential carrier for topical delivery of siRNA: A comparative study of dendriplex vs. lipoplex for delivery of TNF-α siRNA. Int. J. Pharm..

[44] Spagnou S., Miller A.D., Keller M. (2004). Lipidic carriers of siRNA: Differences in the formulation, cellular uptake, and delivery with plasmid DNA. Biochemistry.

[45] Van Gaal E.V.B., van Eijk R., Oosting R.S., Kok R.J., Hennink W.E., Crommelin D.J.A., Mastrobattista E. (2011). How to screen non-viral gene delivery systems in vitro?. J. Control. Release.

[46] Floch V., Loisel S., Guenin E., Herve A.C., Clement J.C., Yaouanc J.J., des Abbayes H., Ferec C. (2000). Cation substitution in cationic phosphonolipids: A new concept to improve transfection activity and decrease cellular toxicity. J. Med. Chem..

[47] Boomer J.A., Thompson D.H., Sullivan S.M. (2002). Formation of plasmid based transfection complexes with an acid-labile cationic lipid: Characterization of in vitro and in vivo gene transfer. Pharm. Res..

[48] Liu D.L., Hu J.J., Qiao W.H., Li Z.S., Zhang S.B., Cheng L.B. (2005). Synthesis of carbamate-linked lipids for gene delivery. Bioorganic Med. Chem. Lett..

[49] Monpara J., Velga D., Verma T., Gupta S., Vavia P. (2019). Cationic cholesterol derivative efficiently delivers the genes: In silicoand in vitro studies. Drug Deliv. Transl. Res..

[50] Zhang Y., Li H., Sun J., Gao J., Liu W., Li B., Guo Y., Chen J. (2010). DC-Chol/DOPE cationic liposomes: A comparative study of the influence factors on plasmid pDNA and siRNA gene delivery. Int. J. Pharm..

[51] Park H.S., Jung H.Y., Park E.Y., Kim J., Lee W.J., Bae Y.S. (2004). Cutting edge: Direct interaction of TLR4 with NAD(P)H oxidase 4 isozyme is essential for lipopolysaccharide-induced production of reactive oxygen species and activation of NF-kappa B. J. Immunol..

[52] Soenen S.J.H., Illyes E., Vercauteren D., Braeckmans K., Majer Z., De Smedt S.C., De Cuyper M. (2009). The role of nanoparticle concentration-dependent induction of cellular stress in the internalization of non-toxic cationic magnetoliposomes. Biomaterials.

[53] Shi J., Yu S., Zhu J., Zhi D., Zhao Y., Cui S., Zhang S. (2016). Carbamate-linked cationic lipids with different hydrocarbon chains for gene delivery. Colloids Surf. B Biointerfaces.

[54] Habib S., Daniels A., Ariatti M., Singh M. (2020). Anti-c-myc cholesterol based lipoplexes as onco-nanotherapeutic agents in vitro. F1000Research.

[55] Bulbake U., Kommineni N., Ionov M., Bryszewska M., Khan W. (2020). Comparison of cationic liposome and PAMAM dendrimer for delivery of anti-Plk1siRNA in breast cancer treatment. Pharm. Dev. Technol..

[56] Zhang L., Mu C., Zhang T., Wang Y., Wang Y., Fan L., Liu C., Chen H., Shen J., Wei K. (2020). Systemic Delivery of Aptamer-Conjugated XBP1 siRNA Nanoparticles for Efficient Suppression of HER2+ Breast Cancer. ACS Appl. Mater Interfaces.

[57] Lee J., Park Y.H. (2022). Trastuzumab deruxtecan for HER2+ advanced breast cancer. Future Oncol..

